# Supplementary material for: Clinical Characteristics of Neurocysticercosis in a Peruvian Population-Based Epilepsy Cohort: A Descriptive Cross-Sectional Study of Baseline Clinical Intake
Source: Pathogens. 2023 Nov 3;12(11):1313. doi: 10.3390/pathogens12111313 (PMC10675766; doi:10.3390/pathogens12111313)

Supplemental Materials

Table S1: Description of cohort follow up

No. enrolled	1975
Date range of first encounter (mm/dd/yyyy)	02/04/2006 – 3/13/2020
Date range of baseline clinical intake visit (mm/dd/yyyy)	01/03/2007 – 03/13/2020
No. with any follow up	1862
No. with at least 1 **clinical follow-up**	1706
Average no. **clinical follow-up** visits (range)	8.4 (1-68)
No. with at least 1 **home visit**	1820
Average no. **home visits** (range)	18.9 (1-138)
Average no. years followed (range)	2.9 (0-13.3)

Figure S1: Length of follow up in years by participants (n=1862)

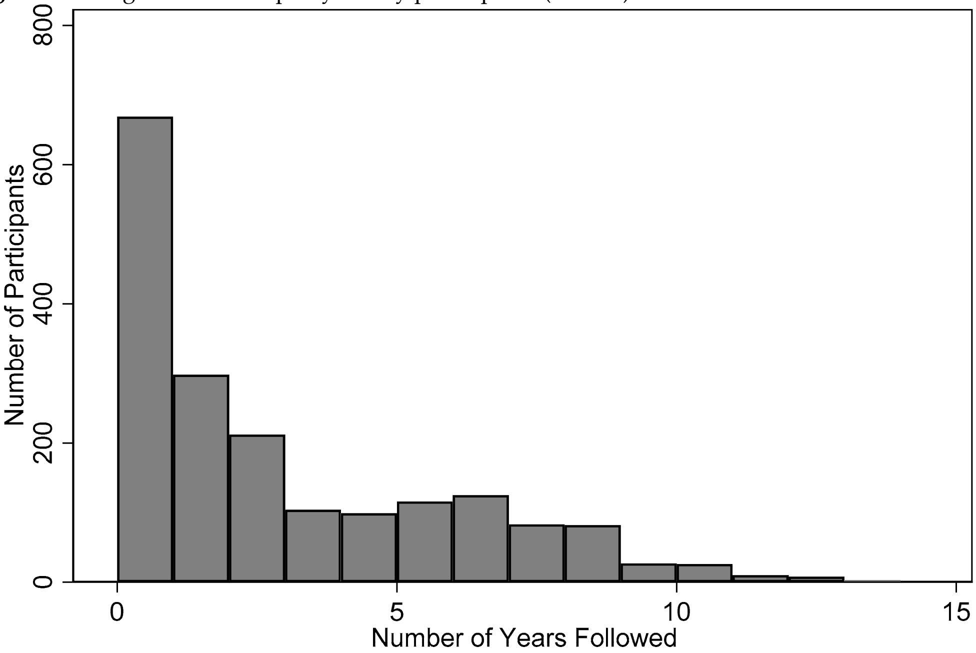

Table S2. Demographic, health, and epilepsy characteristics among the rural Peru epilepsy cohort by neurocysticercosis (NCC) diagnosis overall and stratified by age categories, 2007-2020

	**Total Population**			**Age Categories**						
	**All Ages**			**< 20 years**		**20-39 years**			**40+ years**	
	**N = 1792**			**N = 592**		**N = 704**			**N = 494**	
	**NCC**	**Non-NCC**		**NCC**	**Non-NCC**	**NCC**	**Non-NCC**		**NCC**	**Non-NCC**
	N = 679	N = 1113		N = 151	N = 441	N = 275	N = 429		N = 253	N = 241
Demographics										
**Age (years)**										
Average with SD	34.3 (16.9)	27.0 (16.3)	*	13.8 (3.9)	12.0 (4.9)	28.8 (5.7)	28.8 (5.6)		52.5 (10.6	51.3 (10.6)
**Sex**										
Male	333 (49.0)	545 (49.0)		75 (49.7)	228 (51.7)	138 (50.2)	211 (49.2)		120 (47.3)	105 (43.6)
Female	346 (51.0)	568 (51.0)		76 (50.3)	231 (48.3)	137 (49.8)	218 (50.8)		133 (52.6)	136 (56.4)
Health Characteristics										
**BMI**										
Underweight	11 (1.6)	32 (2.9)	*	7 (4.6)	17 (3.9)	2 (0.7)	8 (1.9)		2 (0.8)	6 (2.5)
Healthy	189 (27.8)	368 (33.1)		49 (32.5)	192 (43.5)	86 (31.3)	109 (25.4)		54 (21.3)	67 (27.8)
Overweight or obese	188 (27.7)	281 (25.3)		25 (16.6)	76 (17.2)	70 (25.5)	119 (27.7)		93 (36.8)	86 (35.7)
Missing	291 (42.9)	432 (38.8)		70 (46.4)	156 (35.4)	117 (42.6)	193 (45.0)		104 (41.1)	82 (34.0)
**Substance Use**										
Tobacco	36 (9.4)	35 (5.3)	*	2 (2.4)	6 (2.2)	21 (13.8)	16 (6.9)		13 (8.8)	13 (8.2)
Alcohol	163 (42.3)	202 (30.2)	*	13 (15.5)	30 (11.0)	88 (57.5)	102 (43.6)	*	62 (41.9)	69 (43.7)
Drugs	1 (0.3)	5 (0.8)		0 (0.0)	3 (1.1)	1 (0.65)	1 (0.4)		0 (0.0)	1 (0.6)
**Medical comorbidities**										
Diabetes	10 (4.6)	12 (3.1)		0 (0.0)	1 (0.6)	2 (2.3)	1 (0.8)		8 (9.1)	10 (10.8)
Hypertension	30 (12.8)	23 (5.8)	*	0 (0.0)	0 (0.0)	3 (3.4)	4 (2.9)		27 (26.2)	19 (19.2)
Dyslipidemia	16 (7.4)	16 (4.3)		0 (0.0)	1 (0.7)	5 (6.0)	5 (3.8)		11 (12.2)	10 (11.6)
Heart Disease	7 (3.4)	6 (1.6)		0 (0.0)	2 (1.3)	2 (2.5)	1 (0.8)		5 (5.8)	3 (3.7)
Psychiatric diagnosis	9 (2.0)	26 (3.5)		3 (3.2)	5 (1.8)	2 (1.2)	15 (5.3)		4 (2.2)	6 (3.5)
**Epilepsy Risk Factors**										
Head Trauma	1 (3.6)	11 (14.7)		0 (0.0)	4 (12.9)	0 (0.0)	4 (14.3)		1 (9.1)	3 (18.8)
FH of epilepsy	380 (62.0)	646 (64.4)		85 (63.4)	287 (70.7)	167 (65.5)	245 (63.3)		128 (57.1)	113 (54.3)

History of neurosurgery	3 (0.5)	8 (0.8)		0 (0.0)	3 (0.8)		2 (0.8)	1 (0.3)		1 (0.4)	4 (1.8)	
Developmental abnormality	51 (10.0)	136 (15.0)	*	11 (8.7)	67 (17.2)	*	20 (10.0)	59 (17.0)	*	20 (10.9)	10 (5.9)	*
Epilepsy Characteristics												
**Active epilepsy***												
Active	572 (88.0)	971 (91.4)		138 (93.2)	401 (94.6)		240 (90.2)	385 (94.4)	*	194 (82.8)	194 (80.0)	
Inactive	78 (12.0)	92 (8.7)	*	10 (6.8)	23 (5.4)		26 (9.8)	23 (5.6)		42 (17.8)	46 (20.0)	
**Age of Onset**												
< 18 years	338 (57.6)	661 (69.3)		122 (96.1)	359 (97.6)		138 (57.7)	228 (59.8)		78 (35.3)	74 (36.1)	
18 or older	249 (42.4)	293 (30.7)	*	5 (3.9)	9 (2.5)		101 (42.3)	153 (40.2)		143 (64.7)	131 (63.9)	
**Duration of Epilepsy**												
< 2 years	111 (18.8)	207 (21.5)	*	45 (34.9)	129 (34.4)		44 (18.4)	47 (12.3)		22 (9.9)	30 (14.6)	
2-5 years	86 (14.6)	211 (21.9)		35 (27.1)	112 (29.9)		31 (13.0)	72 (18.9)		20 (9.0)	27 (13.2)	
6-10 years	82 (13.9)	132 (13.7)		31 (24.0)	74 (19.7)		29 (12.1)	47 (12.3)		22 (9.9)	11 (5.4)	
11-15 years	69 (11.7)	112 (11.6)		14 (10.9)	41 (10.9)		43 (18.0)	59 (15.5)		12 (5.4)	12 (5.4)	
16-20 years	60 (10.2)	91 (9.5)		2 (1.6)	16 (4.3)		38 (15.9)	63 (16.5)		20 (9.0)	12 (5.9)	
20 years	182 (30.9)	210 (21.8)		2 (1.6)	3 (0.8)		54 (22.6)	93 (24.4)		126 (56.8)	113 (55.1)	
**Seizure types by history**												
Generalized	112 (21.3)	250 (28.1)	*	23 (19.3)	111 (31.9)	*	47 (21.9)	82 (24.2)		42 (21.9)	56 (28.1)	
Focal	397 (75.5)	623 (70.0)		90 (75.6)	228 (65.5)		163 (75.8)	252 (74.3)		144 (75.0)	139 (69.9)	
Indeterminate	17 (3.2)	17 (2.0)		6 (5.0)	9 (2.6)		5 (2.3)	5 (1.5)		6 (3.1)	4 (2.0)	
**Reported any current/past ASM usage**												
Yes	134 (19.7)	279 (25.1)	*	29 (19.2)	106 (24.0)		54 (10.6)	114 (26.6)	*	51 (20.2)	59 (24.5)	

* Indicates P-values < 0.05

NB: percentages are reported among available data

Table S3. Demographic and epilepsy characteristics among the epilepsy cohort by neurocysticercosis (NCC) diagnosis overall and stratified by active epilepsy, 2007-2020

	**Total**			**Active Epilepsy**			**Inactive Epilepsy**			**Missing**		
	**N = 1792**			**N = 1543**			**N = 170**			**N = 79**		
	**NCC**	**Non-NCC**		**NCC**	**Non-NCC**		**NCC**	**Non-NCC**		**NCC**	**Non-NCC**	
	N = 679	N = 1113		N = 572	N = 971		N = 78	N = 92		N = 29	N = 50	
Demographics												
Age	34.3 (16.9)	27.0 (16.3)		32.9 (16.4)	26.0 (15.6)		41.9 (18.5)	36.9 (19.6)		42.3 (16.4)	29.0 (16.8)	
Sex (male)	333 (49.0)	545 (49.0)		276 (48.3)	475 (48.9)		43 (55.1)	41 (44.6)		14 (48.3)	29 (58.0)	
Epilepsy Characteristics												
**Age of Onset**												
18 or older	249 (42.4)	293 (30.7)	*	212 (42.4)	264 (31.1)	*	30 (41.1)	25 (31.7)		7 (50.0)	4 (15.4)	*
**Duration of Epilepsy**												
< 2 years	111 (18.8)	207 (21.5)	*	110 (21.9)	203 (23.7)	*	1 (1.4)	3 (3.8)		0 (0.0)	1 (3.7)	
2-5 years	86 (14.6)	211 (21.9)		82 (16.3)	202 (23.6)		4 (5.5)	8 (10.0)		0 (0.0)	1 (3.7)	
6-10 years	82 (13.9)	132 (13.7)		70 (13.9)	117 (13.7)		10 (13.7)	11 (13.8)		2 (14.3)	4 (14.8)	
11-15 years	69 (11.7)	112 (11.6)		57 (11.3)	94 (11.0)		10 (13.7)	13 (16.3)		2 (14.3)	5 (18.5)	
16-20 years	60 (10.2)	91 (9.5)		52 (10.3)	78 (9.1)		6 (8.2)	10 (12.5)		2 (14.3)	3 (11.1)	
20+ years	182 (30.9)	210 (21.8)		132 (26.2)	162 (18.9)		42 (57.5)	35 (43.8)		8 (57.1)	13 (48.2)	
**Seizure types by history**												
Generalized	112 (21.3)	250 (28.1)	*	76 (17.0)	203 (25.6)	*	36 (47.4)	45 (49.5)		0	1	
Focal	397 (75.5)	623 (70.0)		357 (79.7)	574 (72.4)		39 (51.3)	44 (48.4)		1	1	
Indeterminate	17 (3.2)	18 (2.0)		15 (3.4)	16 (2.0)		1 (1.3)	2 (2.2)		1	0	
**EEG Result**												
Normal	35 (46.7)	60 (36.8)		33 (45.8)	53 (35.6)		0	1				
Non-specific abnormality	20 (26.7)	49 (30.1)		20 (27.8)	46 (30.9)		0	1				
Focal epileptiform	12 (16.0)	21 (12.9)		12 (16.7)	18 (12.1)		0	0				
Generalized epileptiform	3 (4.0)	16 (9.8)		2 (2.8)	16 (10.7)		0	0				
Epileptiform uncertain focal versus generalized	5 (6.7)	7 (10.4)		5 (6.9)	16 (10.7)		0	0				
**Reported current/past ASM usage**												
Yes	134 (19.7)	279 (25.1)		131 (22.9)	266 (27.4)		0	3 (3.3)		3 (10.3)	10 (20.0)	
**ASM prescribed at baseline**												
Yes	539 (87.4)	859 (87.7)		539 (97.6)	859 (99.6)		NA	NA		0	0	
Cysticercosis Diagnostic Serologies												
EITB assay results												
1 or more	359 (56.1)	234 (23.9)	*	295 (54.9)	192 (22.6)	*	49 (63.6)	31 (35.2)	*	15 (57.7)	11 (27.5)	
Antigen ELISA results												

Positive	41 (41.0)	8 (14.0)	*	35 (39.8)	7 (14.9)		5 (50.0)	1 (14.3)	1 (50.0)	0 (0.0)

* Indicates P-values < 0.05

NB: percentages are reported among available data

Table S4. Enzyme-linked immunoelectrotransfer blot (EITB) for *Taenia solium* results by any NCC and type of NCC diagnosed by computed tomography (CT) scan at baseline clinical intake, 2007-2020

	Calcified lesions, only	Any active cysts	No NCC
	n = 550	n = 90	n = 979
**Positive Western Blot (WB)**			
Any bands	291 (53.1)	67 (74.4)	234 (23.9)
**Categorical WB**			
No bands	258 (46.9)	23 (25.6)	745 (76.1)
1-2 bands	86 (15.6)	9 (10.0)	110 (11.2)
3 + bands	206 (37.5)	58 (64.4)	124 (12.7)
**Full WB results**			
No bands	258 (46.9)	23 (25.6)	745 (76.1)
1 band	47 (8.6)	3 (3.3)	64 (6.5)
2 bands	46 (8.4)	7 (7.8)	51 (5.2)
3 bands	114 (20.7)	20 (22.2)	96 (9.8)
4 bands	26 (4.7)	6 (6.7)	9 (0.9)
5 bands	11 (2.0)	3 (3.3)	5 (0.5)
6 bands	11 (2.0)	5 (5.6)	3 (0.3)
7 bands	37 (6.7)	23 (25.6)	6 (0.6)

All differences in proportions for WB results by NCC categories were statistically significant ($P < 0.0001$)

Figure S2: Stacked Bar Plot of Cumulative Percentages by NCC Imaging Categories

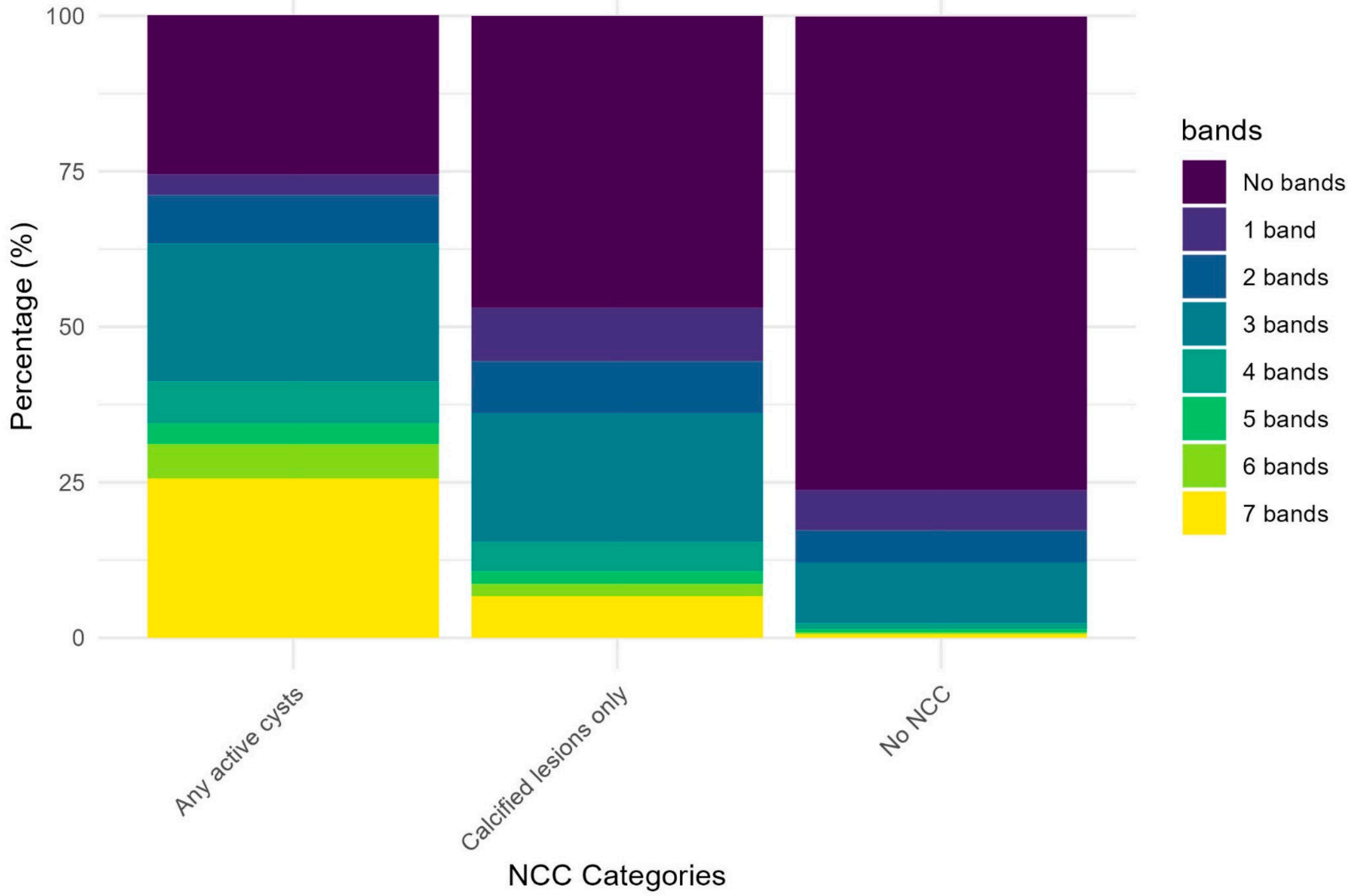

Supplement: Supplementary file 1 [file pathogens-12-01313-s001.zip › pathogens-2666266-supplementary.pdf]
